# Medical Reform

**Published:** 1877-10

**Authors:** 


*Some General Ideas concerning Medical Reform.* By DAVID HUNT, M. D. Boston: A. Williams and Company. New York: Wm. Wood and Co., 1877. pp. 50.

This is an essay upon the growth of medicine, with the last few pages devoted to the discussion of the status of the medical profession with the mode of procedure of raising it and more thoroughly educating the applicants for the degree of M. D. The ideas advanced are sound. Hear what he says concerning the aim of medical education: "The truth that the aim of medical education should be in the greater part to form a good observer, and not a well-crammed graduate, needs to be emphasized. It is better to fit the student to select from the mass of medical literature of the day, than to incite him to attempt an acquaintance with it all. To this end, teachers should be critical observers; students learn much by imitating their teachers." And again: "Train our students to observe; select tutors from the world, not from a city or street; let every man in the State or country feel that good work is a sure passport, that nothing else avails; and the results of trained observation will nourish the drooping plant that now causes so much solicitude." The whole pamphlet is well written and will be found entertaining reading. C.

